# Genome-wide association study for variants that modulate relationships between cerebrospinal fluid amyloid-beta 42, tau, and p-tau levels

**DOI:** 10.1186/s13195-018-0410-y

**Published:** 2018-08-28

**Authors:** Taylor J. Maxwell, Chris Corcoran, Jorge L. del-Aguila, John P. Budde, Yuetiva Deming, Carlos Cruchaga, Alison M. Goate, John S. K. Kauwe, Alzheimer’s Disease Neuroimaging Initiative

**Affiliations:** 1 Computational Biology Institute, The George Washington University, 45085 University Drive, Suite 305, Ashburn, VA 20148 USA; 2 Department of Mathematics and Statistics, Utah State University, 3900 Old Main Hill, Logan, UT 84322 USA; 3 Department of Psychiatry, Washington University School of Medicine, 660 S. Euclid Ave. B8134, St. Louis, MO 63110 USA; 4 Department of Genetics and Genomic Sciences, Icahn School of Medicine at Mount Sinai, 1425 Madison Avenue, ICAHN 10-52, New York, NY 10029 USA; 5 Ronald M. Loeb Center for Alzheimer’s Disease, Department of Neuroscience, Icahn School of Medicine at Mount Sinai, 1425 Madison Avenue, ICAHN 10-52, New York, NY 10029 USA; 6 Department of Biology, Brigham Young University, 4146 LSB, Provo, UT 84602 USA

**Keywords:** rQTL, G×G, Alzheimer’s, AΒ42, Tau, p-tau (3 to 10)

## Abstract

**Background:**

A relationship quantitative trait locus exists when the correlation between multiple traits varies by genotype for that locus. Relationship quantitative trait loci (rQTL) are often involved in gene-by-gene (G×G) interactions or gene-by-environmental interactions, making them a powerful tool for detecting G×G.

**Methods:**

We performed genome-wide association studies to identify rQTL between tau and *Aβ42* and *ptau* and *Aβ42* with over 3000 individuals using age, gender, series, *APOE* ε*2*, *APOE* ε*4*, and two principal components for population structure as covariates. Each significant rQTL was separately screened for interactions with other loci for each trait in the rQTL model. Parametric bootstrapping was used to assess significance.

**Results:**

We found four significant *tau*/*Aβ42* rQTL from three unique locations and six *ptau*/*Aβ42* rQTL from five unique locations. G×G screens with these rQTL produced four significant G×G interactions (one *Aβ42*, two *ptau*, and one *tau*) with four rQTL where each second locus was from a unique location. On follow-up, rs1036819 and rs74025622 were associated with Alzheimer’s disease (AD) case/control status; rs15205 and rs79099429 were associated with rate of decline.

**Conclusions:**

The two most significant rQTL (rs8027714 and rs1036819) for *ptau*/*Aβ42* are on different chromosomes and both are strong hits for pelvic organ prolapse. While diseases of the nervous system can cause pelvic organ prolapse, it is unlikely related to the *ptau*/*Aβ42* relationship but may suggest that these two loci share a pathway. In addition to a *ptau/Aβ42* rQTL and association with AD case/control status, rs1036819 is a strong rQTL for case/control status/*Aβ42* and for *tau/Aβ42*. It resides in the *ZFAT* gene, which is related to autoimmune thyroid disease. For *tau*, rs9817620 interacts with the *tau*/*Aβ42* rQTL rs74025622. It is in the *CHL1* gene, which is a neural cell adhesion molecule and may be involved in signal transduction pathways. *CHL1* is related to *BACE1*, which is a β-secretase enzyme that initiates production of the β-amyloid peptide involved in AD and is a primary drug target. Overall, there are numerous loci that affect the relationship between these important AD endophenotypes and some are due to interactions with other loci. Some affect the risk of AD and/or rate of progression.

**Electronic supplementary material:**

The online version of this article (10.1186/s13195-018-0410-y) contains supplementary material, which is available to authorized users.

## Background

*Aβ42*, *tau*, and *ptau* are important biomarkers for Alzheimer’s disease (AD). Cerebrospinal fluid (CSF) levels of amyloid-beta (Aβ) and tau change before clinical symptoms of AD are observed. Aβ42 is decreased in CSF as the disease progresses, and tau levels increase. This combination of changes appears to be specific to AD [1]. CSF levels of Aβ42 and tau are important indicators of AD pathology. Here, we seek to identify genetic loci that control these levels and modify the relationships between them. A better understanding of the genes and pathways that regulate the relationships between these biomarkers may provide important insights into AD pathology, even at the earliest stages of the disease. Many large genome-wide association studies (GWAS) have been successful in finding loci that affect AD biomarkers such as *Aβ42*, *tau*, and *ptau*. In a series of papers, Hohman et al. [2–4] performed a variety of analyses that screened for loci that modify the relationship between various AD-related risk factors. More formally, loci that affect the relationship between two or more traits can be referred to as relationship loci or relationship quantitative trait loci (rQTL) [5]. Differential gene-by-gene (G×G) and/or gene-by-environment (G×E) interactions are often responsible for rQTL [6]. Screening for rQTL is a way to identify important loci that would typically be “invisible” to normal GWAS because they often do not show marginal effects on either trait [7]. It is also a powerful avenue to identify G×G interactions without paying the statistical penalty of performing all possible two-locus tests. rQTL also provide a window into pleiotropy, pleiotropic variability, and selection on traits in complex interconnecting systems [8].

Here, we present the results of genome-wide screens for rQTL using CSF levels of *Aβ42*, *tau*, and *ptau* subsequent screens for loci that interact with them (G×G). We then present analyses designed to connect the significant rQTL and G×G loci from the primary analyses with AD itself, first by testing the hypothesis that if a locus modulates the relationship between two AD biomarkers (i.e., an rQTL) it may modulate the risk relationship between AD and either biomarker (i.e., an AD/biomarker rQTL), and finally we follow-up with associations of these markers with AD risk and rate of progression (rate of cognitive decline).

These analyses are designed to identify context-dependent effects that are often not “seen” in traditional marginal effect screens (i.e., a typical GWAS). We demonstrate that they exist and that they may point us to other pathways. While interactions can be hard to detect and interpret, they are expected in complex biological systems and they are important as they can create unexplained heterogeneity and can identify subgroups that respond differently to treatment (G×E) or in the context of other genes (G×G).

## Methods

The data and analyses can be grouped into primary analyses and follow-up. The primary analyses (rQTL and G×G screens) use a large CSF AD biomarker dataset (*n* = 3146) derived from nine separate studies. The follow up analyses use different datasets to follow-up on the loci found in the primary rQTL and G×G screens. There are three follow-up analyses. The first uses the original CSF biomarker data to determine if any of the significant biomarker rQTL act as rQTL between disease (AD risk) and either biomarker. In the second follow-up, the loci were tested for association with AD risk using a large AD case/control dataset (*n* = 28,730) from the Alzheimer’s Disease Genetic Consortium (ADGC) and secondarily with results from the International Genomics of Alzheimer’s Project (IGAP) consortium (*n* = 54,162). The last follow-up is based on the association with rate of progression (cognitive decline) which was performed using data (*n* = 1499) from the Alzheimer’s Disease Neuroimaging Initiative (ADNI) and the Charles F. and Joanne Knight Alzheimer’s Disease Research Center (Knight ADRC) from Washington University in St. Louis, USA. Direct replication of these results is not possible at this time as there are no other CSF AD biomarker datasets sufficient for replication; we hope to remedy this in the future. Our best approach akin to replication is to establish a connection between these loci and relevant AD endpoints such as risk and rate of progression.

### Data and methods for the rQTL and G×G analyses

For the rQTL and G×G analyses, the dataset for CSF biomarker analysis consisted of 3146 participants from nine different studies and is described in detail in our recent publication [9]. Included were 805 individuals (29.34% cases) enrolled in studies at the Knight ADRC, 787 individuals (more than 71% cases) from the ADNI (390 from ADNI1 and 397 from ADNI2), 184 individuals (5.43% cases) from Predictors of Cognitive Decline Among Normal Individuals (BIOCARD), 105 individuals (no AD status) from Saarland University in Homburg/Saar, Germany, 433 individuals (22.17% cases) from the Mayo Clinic, 293 individuals (all cases) from Skåne University Hospital, Sweden, 164 (62.8% cases) from studies at Perelman School of Medicine at the University of Pennsylvania, and 375 (33.33% cases) from studies at the University of Washington. Table 1 shows the demographic data for each study. Clinical assessments, CSF collection, and proteins were measured by each site. Prior to combining data for analyses, CSF levels of tau, p-tau, and Aβ42 were log_10_-transformed to approximate a normal distribution and the mean from each dataset was standardized to zero to account for the different platforms used by the different studies to measure protein levels. There were no significant differences in the transformed and standardized values for the different studies. Study, age, sex, and the first two principal components were identified as confounding factors by stepwise regression analyses for each protein and corrected for in applicable analyses [9]; other papers have been published that have combined data in similar ways [10, 11].

**Table 1:** Cohort demographics

	Knight ADRC	ADNI1	ADNI2	BIOCARD	HB	Mayo	Sweden	UPenn	UW
*n* = 3146	805	390	397	184	105	433	293	164	375
Age (years)	70.39 ± 9.12	77.89 ± 6.89	73.28 ± 7.47	62.10 ± 9.46	67.52 ± 9.24	78.73 ± 6.35	75.15 ± 7.63	71.60 ± 8.98	62.35 ± 16
Age range (years)	37–91	58–93	55–92	23–86	45–84	50–95	50–88	50–94	21–88
Male (%)	46.09	60	54.91	41.53	54.29	60.51	37.54	41.46	50.67
APOE ε4 positive (%)	40.75	50	38.29	34.43	54.29	27.5	76.11	55.56	43.28
CDR > 0 (%)	29.34	71.28	71.03	5.43	–	22.17	100	62.8	33.33
Aβ_42_ levels^a^	650.40 ± 305.59	169.83 ± 56.0	179.98 ± 51.31	386.90 ± 89.93	77.59 ± 23.30	331.0 ± 122.21	262.43 ± 72.77	163.55 ± 53.54	141.90 ± 41.42
p-tau_181_ levels^a^	64.94 ± 34.26	34.13 ± 18.52	38.63 ± 21.21	38.94 ± 12.30	–	23.16 ± 10.55	105.76 ± 41.82	36.96 ± 26.80	56.56 ± 29.32
tau levels^a^	372.40 ± 235.41	97.26 ± 52.03	79.69 ± 47.79	66.56 ± 26.60	84.27 ± 36.79	104.29 ± 58.06	782.20 ± 301.68	93.66 ± 54.29	61.64 ± 42.77

^a^Reported as mean ± standard deviation in pg/mL

*ADRC* Alzheimer’s Disease Research Center, *ADNI* Alzheimer’s Disease Neuroimaging Initiative, *APOE* apolipoprotein E, *BIOCARD* Predictors of Cognitive Decline Among Normal Individuals, *CDR* Clinical Dementia Rating, *HB* Saarland University in Homburg/Saar, Germany, *Mayo* Mayo Clinic, *Sweden* Sahlgren’s University Hospital, Sweden, *UPenn* Perelman School of Medicine at the University of Pennsylvania, *UW* University of Washington

### rQTL and G×G genetic data: for the rQTL and G×G analyses

Five million single nucleotide polymorphisms (SNPs) (5,088,365) were included for analyses from the 12 million available imputed SNPs after excluding those with a minor allele frequency (MAF) less than 0.01. Data was imputed using the 1000 Genomes Phase 3 data (October 2014) as the reference panel [9]. For the rQTL screens we used a standard 5 × 10^−8^ genome-wide significance threshold and for the G×G screens we corrected for 5 × 10^−8^ divided by the number of independent and significant rQTL from each screen (*tau/Aβ42* and p*tau/Aβ42*; see Table 3). Most analyses were performed using the Julia programming language (v0.3.12) [12, 13] and some plots were made in R [14].

### rQTL analyses

As a strategy to detect G×G interactions, we performed genome-wide single-locus screens for rQTL [4] with the CSF biomarker data. All models used the same covariates: age, sex, series, APOε2, APOε4, and the first two principle components for ancestry. Significant rQTL were used as independent a-priori hypotheses to identify G×G interactions. Two rQTL models were screened:

1 $$ tau=u+\mathrm{covariates}+ A\beta 42+ SNP+ A\beta 42\ast SNP $$

2 $$ ptau=u+\mathrm{covariates}+ A\beta 42+ SNP+ A\beta 42\ast SNP $$

where the SNP genotypes are treated as factors and only genotypes with a count of 20 or more were included, leading to models with sufficient genotype counts for tests; either a three-genotype model with 2 degrees of freedom (DF) or a two-genotype model with 1 DF. A putative rQTL exists when the Aβ42*SNP interaction term is significant when compared with a null model excluding that term. Tests of interaction terms sometimes exhibit type I error inflation due to underestimates of the covariance matrix [15]. Work performed by Bůžková et al. [16] and Voorman et al. [14] showed that sandwich estimators and the parametric bootstrap can be used to obtain valid *p* values, with the parametric bootstrap as the gold standard. For tests that initially reached the significance threshold, empirical *p* values were obtained via 200 million parametric bootstraps [5]. A standard 5 × 10^−8^ genome-wide significance threshold was used for each rQTL screen.

### G×G analyses

Context-dependent interactions such as G×E and G×G create single locus patterns such as rQTL. Therefore, each significant rQTL create independent a-priori hypotheses for G×E or G×G interactions. Previous papers on two-stage designs for G×G and G×E studies have shown that each a-priori locus (i.e., loci found in single locus screens) can be treated separately for multiple testing in genome screens for interaction [17–21]. For each significant rQTL, we performed separate screens for loci that interact (G×G) with each rQTL and their specific traits. We corrected for 5 × 10^−8^ divided by the number of independent and significant rQTL from each screen (*tau/Aβ42* and p*tau/Aβ42*).

Traditionally, in human genetics tests of G×G focus only on the additive-by-additive (AA) contrast while interactions between two biallelic loci can have up to four “orthogonal” contrasts that are traditionally parameterized as AA, additive-by-dominance (AD), dominance-by-additive (DA), and dominance-by-dominance (DD). These other types of interactions exist and would be missed with only an AA test. Unfortunately, in natural populations, allele frequencies are not 50/50 which results in unbalanced and sometimes missing cells (two-locus genotypes). The unbalanced cell counts makes the “orthogonal” contrasts no longer orthogonal and sometimes inestimable when there are missing cells [22–24]. We used a simple interaction test that avoids the need for explicit parameterization of the interaction contrasts and only accounts for interactions with sufficient data for estimation. Our full model incorporates covariates and treats the two-locus genotypes as factors similar to a one-way analysis of covariance (ANCOVA). The reduced model includes the covariates and treats the genotypes from each individual locus as factors [25]. The full model accounts for all single and two-locus interaction effects without explicitly parameterizing them while the reduced model explicitly accounts for just the single locus effects. With sufficient counts for all two-locus genotypes the test has 4 DF. All two-locus genotypes with counts less than five were excluded which ensures that estimation of the interaction effect is appropriate while avoiding the issues of inestimable contrasts or trying to determine explicit contrasts for each particular case. Below is an example of the full model:

3 $$ trait=u+ covariates+ TwoLocGenotypes $$

where the two-locus genotypes are treated as factors, and below is an example of the reduced model where the genotypes of each SNP are treated as factors:

4 $$ trait=u+ covariates+ rQTL+ SNP $$

Parametric bootstrapping [5, 16] was applied to any test that initially met the significance threshold with 100 million replicates.

### Follow-up

We attempted to connect the significant rQTL and G×G loci identified in the primary biomarker analyses to AD etiology in three different ways. First, we determined if each rQTL between biomarkers acts as an rQTL between AD risk and either of the two biomarkers. This hypothesis extends from the observation that if a locus affects the relationship between two risk factors, it may also modify the relationship between those risk factors and disease. Therefore, each rQTL was fitted for an analogous rQTL model using logistic regression between Alzheimer’s case/control status and that risk factor using the same CSF biomarker data. Second, we used a large AD case/control dataset to assess whether any of the rQTL or G×G loci are directly associated with AD risk. Finally, we use a different dataset to assess if any of these loci are associated with disease progression via rate of cognitive decline.

### Data (ADGC and IGAP) and methods for AD risk analyses

We used two datasets for connecting the loci to AD risk through AD case/control data. We used the ADGC as our primary analyses but also report the results from IGAP. While the IGAP study is larger (it includes ADGC), we feel that the ADGC data alone is more homogenous and is a closer match in terms of ethnic composition to our discovery dataset. The ADGC is an NIH-funded collection of GWAS data created for the goal of identifying genetic contributions to late-onset AD. Participants included in this study were from 30 merged datasets [26] combined by Boehme et al. [27] and included 25,666 unrelated individuals carrying either an AD (*n* = 12,532) or cognitively normal control clinical diagnosis (*n* = 13,134). Participants were recruited and seen between 1984 and 2012. The genetic data are based on merging the different imputed ADGC datasets. Before merging, SNPs within each dataset with low imputation quality (info < 0.5) were filtered out. Dosage information was used to convert to best guess PLINK allele call format files with an uncertainty cutoff of 0.1. Duplicate samples were identified and removed. Other quality control issues such as physical location discrepancies and strand flipping were identified and appropriately dealt with. A kinship coefficient of 0.0442 was used to screen out third-degree relatives or closer. The resulting number of SNPs in the unrelated dataset was ~ 8.6 million. We only used the SNPs corresponding to our significant rQTL and G×G loci, as simple logistic regression analysis was performed to assess the association between our SNPs and case control status using age, sex, cohort, and two principle components as covariates. Full details on the datasets and the merging process are available at [27].

The IGAP consortium organized AD case/control data from many different studies across the world. For stage 1 of their analysis they used meta-analysis methods for the association between ~ 7 million SNPs and AD case/control data (*n* = 54,162; 17,008 cases, 37,154 control) from four studies (ADGC, CHARGE, EADI, and GERAD) [28]. We used the results from the stage 1 analyses to determine whether any of the significant rQTL or G×G loci are associated with AD risk. In the preparation of the genetic data, Lambert et al. [28] excluded SNPs with call rates < 95%, imputed using samples of European ancestry in the 1000 Genome Project, and excluded SNPs with MAF < 1%. In each case/control dataset, the association of late-onset Alzheimer’s disease with genotype dosage was analyzed by a logistic regression model including covariates for age, sex, and principal components to adjust for possible population stratification. For the three CHARGE cohorts with incident Alzheimer’s disease data, Cox proportional hazards models were used. For the meta-analysis they undertook a fixed-effects inverse variance-weighted meta-analysis with the standard errors of the beta coefficient scaled by the square roots of study-specific genomic inflation factors estimated before combining the summary statistics across datasets [28].

About half of the samples from the CSF AD biomarker data can be found in both the ADGC and IGAP datasets and only represent ~ 5.5% and ~ 3% of the ADGC and IGAP samples. It is also important to note that the analyses leading to these follow-up tests for association with case/control status in ADGC and IGAP are based on rQTL and G×G analyses using *Aβ42*, *tau*, and *ptau* and not any explicit single-locus test for association with case/control status in the CSF samples.

### Data and methods for rate of progression analyses

A total of 1499 individuals with longitudinal cognitive data and genetic data were used to assess the association of the significant rQTL and G×G loci with rate of progression or the rate of cognitive decline. More extensive descriptions of the data and methods are described elsewhere [29]. Participants from this study were enrolled in two different longitudinal studies: the Knight ADRC at Washington University (*n* = 778) and the ADNI (*n* = 712). Participants were evaluated in accordance with the Clinical Dementia Rating (CDR) scale, where 0 indicates cognitive normality, 0.5 is very mild dementia, 1 is mild dementia, 2 is moderate dementia, and 3 is severe dementia [30, 31]. The scores in each of the six areas are summed to yield a sum of box (CRD-SB) scores ranging from 0 (no impairment) to 18 (maximal impairment). Only participants that had an AD diagnosis and CDR > 0 at their last visit were included in our analyses. Individuals with dementia caused by neurological diseases other than AD were excluded. For those samples with data available, individuals with Aβ42 values equal or greater than 192 pg/mL (ADNI) [32] or 500 pg/mL (WU) [33] were also excluded. Noninformative longitudinally measured CDR-SB was removed for each participant (noninformative longitudinal data is defined as data where the CDR-SB is either 0 or 18 and remains constant over a period of time). Only individuals with at least two visits and 1.5 years of follow-up were included.

Participants were genotyped with the Illumina 610 or Omniexpress chip (Illumina, San Diego, CA, USA). IMPUT2 v2.3.2 software and the 1000 genome (phase3 NCBI build 37) data were used as reference to impute up to 6 million SNPs. To avoid the possibility of spurious associations of population structure and to confirm ethnicity of each sample, the two principal components scores were used as covariates in the analysis. Only individuals that clustered with the European-American cluster were included in the study.

A linear mixed-model analysis was carried out using R statistical software [14] and the nlme package [34]. A linear mixed-model repeated measure framework was used to account for correlation between repeated measures in the same individual. Change in CDR-SB per year was treated as the independent variable including the following covariants: baseline CDR, baseline age, gender, time (follow-up), level of education, the interaction between baseline CDR and time, and, to avoid the possibility of spurious association due to population substructure, the two first principal components scores were included as covariates, and a random effect for time and individual was included in the model with an AR(1) covariance structure.

## Results

**Fig. 1:**
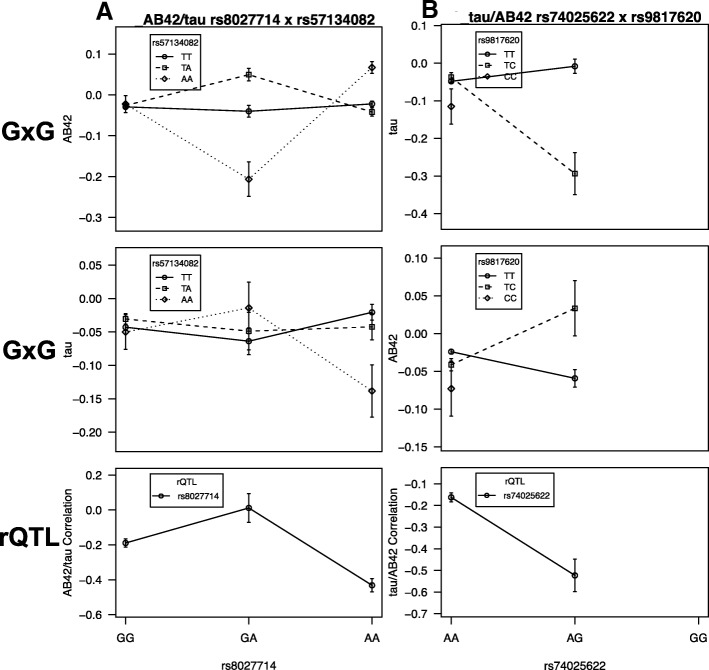
Each column plots (**a** and **b**) the relationship quantitative trait loci (rQTL) pattern and two gene-by-gene (G×G) interaction patterns (one for each trait from the original rQTL model) between the rQTL and a specific SNP. The trait for which the rQTL×SNP interaction was found significant in the screen (Table 2) is the top plot. The middle G×G plot was not found in the G×G screens but is displayed to show the differential two-locus genotypic mean patterns between the traits and how they relate to the rQTL pattern. rQTL are often the result of G×G patterns that are differential across traits. The *x* axis for all plots in a column refers to the genotypes of that specific rQTL. The bottom plot displays the rQTL pattern, which is the covariate-adjusted correlation between the two traits within each genotype of the rQTL along with error bars based on SE from bootstrapping. The G×G plots display the covariate-adjusted two-locus genotypic means for that particular trait (*y* axis) along with SE error bars based on bootstrapping. **a** The rs8027714 *tau*/Aβ42 rQTL interaction with rs57134082 for amyloid-beta (Aβ)42 (top plot) (Table 3) and under nominal for tau (middle; *p* = 0.088). Note the differential pattern between the two interaction plots although it is not clear how they relate to the rQTL pattern. **b** The *tau*/Aβ42 rQTL rs74025622 interactions with rs9817620 for *tau* (top plot) (Table 2) and Aβ42 (middle; *p* = 0.0013). Here, the differential G×G patterns for *tau* and Aβ42 directly result in changing the *tau*/Aβ42 correlations across the rQTL genotypes. For tau, the rs9817620 TC genotype goes from high to low across the rs74025622 genotypes (from AA to AG) while the opposite is true for Aβ42. This corresponds to a much more negative correlation between tau and Aβ42 in the rs74025622 AG genotype. rs9817620 is in the CHL1 gene (see Discussion)

From our rQTL analyses we identified four genome-wide significant *tau/Aβ42* rQTL from three unique locations and six *ptau/Aβ42* rQTL from five unique locations (Table 2). Using the significant rQTL (one from each unique location) as a priori hypotheses, subsequent G×G screens identified loci from four different regions involved in significant two-locus interactions with four of the rQTL (one *Aβ42*, one *tau*, and two *ptau*) (Table 3, Fig. 1) (see “Data and methods for the rQTL and G×G Analyses” in Methods). The respective threshold for G×G screens with the *tau/Aβ42* rQTL was *p* < 1.67 × 10^−8^ and *p* < 1 × 10^−8^ for G×G screens with the *ptau/Aβ42* rQTL.

**Table 2:** Table of genome-wide significant rQTL *p* values based on 200 million parametric bootstraps

Trait pair	SNP	Chrom	Location	Maj/Min (MAF)	*p* value	Gene	Left gene	Right gene
tau/Aβ42	rs74131475	1	194252450	C/T (0.089)	3.33 × 10^–8^	LOC107985242	EEF1A1P14	RNU6-983P
tau/Aβ42	rs1936361	10	102370841	C/T (0.444)	5.00 × 10^–9^		HIF1AN	PAX2
tau/Aβ42	rs118023102	16	75983693	G/A (0.027)	3.33 × 10^–8^		LOC105371348	LOC105371349
tau/Aβ42	rs74025622^a^	16	75999881	A/G (0.028)	< 5 × 10^–9^		LOC105371348	LOC105371349
p-tau/Aβ42	rs15205^b,c^	3	44966906	A/T (0.028)	< 5 × 10^–9^	ZDHHC3	TGM4	EXOSC7
p-tau/Aβ42	rs79099429^b,c^	3	44993764	T/C (0.029)	< 5 × 10^–9^	ZDHHC3	TGM4	EXOSC7
p-tau/Aβ42	rs689167	6	52785560	G/A (0.362)	< 5 × 10^–9^		GSTA3	GSTA9P
p-tau/Aβ42	rs1036819^a^	8	135611945	A/C (0.261)	< 5 × 10^–9^	ZFAT	LOC100129104	LOC286094
p-tau/Aβ42	rs112959610	13	57488227	G/A (0.465)	2.67 × 10^–8^		RN7SKP6	PRR20A
p-tau/Aβ42	rs8027714	15	24964597	G/A (0.201)	< 5 × 10^–9^		C15orf2	SNRPN

*Chrom* Chromosome, *MAF* minor allele frequency, *Maj* Major Allele, *Min* Minor Allele, *rQTL* relationship quantitative trait loci, *SNP* single nucleotide polymorphism

^a^Nominally significant for case/control status

^b^Nominally significant for rate of decline

^c^Nominally significant for Aβ42

**Table 3:** Table of genome-wide significant G×G interactions with rQTL

Trait	rQTL	SNP2	Chrom	Location	Maj/Min (MAF)	*p* value	Gene	Left gene	Right gene
Aβ42	**rs8027714**	**rs57134082**	12	40057892	T/A (0.166)	**< 5 × 10** ^**–9**^	C12orf40	ABCD2	SLC2A13
tau	rs74025622	rs9817620 ^b^	3	261440	T/C (0.062)	1.50 × 10 ^–8^	CHL1	LOC642891	LOC402123
tau	rs74025622	rs73105331^a,b^	7	52374812	C/T (0.031)	3.00 × 10^–8^		LOC107986796	LOC107986738
tau	rs74025622	rs75034965^b^	22	35710231	G/A (0.144)	3.75 × 10^–8^	TOM1	HMGXB4	HMOX1
p-tau	**rs689167**	**rs1558634**	7	29553339	C/T (0.125)	**1.00 × 10** ^**–8**^	CHN2	NANOGP4	LOC646745
p-tau	**rs79099429** ^**a,c**^	**rs79688703** ^**b**^	16	7899349	T/C (0.059)	**5.00 × 10** ^**–9**^		RBFOX1	LOC105371069
p-tau	rs15205^a,c^	rs79688703^b^	16	7899349	T/C (0.059)	2.75 × 10^–8^		RBFOX1	LOC105371069

*p* values based on 100 million parametric bootstraps

SNPs and *p* values in bold are significant after correcting for the number of independent and significant *ptau/Aβ42* rQTL (*p* < 1 × 10^−8^); those underlined are significant after correcting for the *tau/Aβ42* rQTL (*p* < 1.67 × 10^−8^); the rest are not mentioned in the text and are suggestive

*Chrom* Chromosome, *G×G* gene-by-gene, *MAF* minor allele frequency, *Maj* Major Allele, *Min* Minor Allele, *rQTL* relationship quantitative trait loci, *SNP* single nucleotide polymorphism

^a^Nominally significant for rate of decline

^b^Nominally significant for Aβ42 G×G

^c^Nominally significant for direct association with Aβ42

In follow-up, two *ptau/Aβ42* rQTL were significantly associated with AD case/control status in the ADGC dataset (rs1036819, *p* = 1.9 × 10^−5^; rs74025622, *p* = 0.044); however, they were not significant in IGAP (rs1036819, *p* = 0.091; rs74025622, *p* = 0.427) (see the first few paragraphs of “Follow-up” in Methods). In addition, rs1036819 is also a very strong rQTL for both AD case/control status/*Aβ42* (*p* = 6.2 × 10^−8^) and *tau/Aβ42* (*p* = 6.17 × 10^−5^), while rs74025622 is involved in G×G interactions with three other loci (Table 3) (see the first paragraph of “Follow up” in Methods). While not significant in ADGC, rs1558634 (a *ptau* G×G locus; Table 3) is significantly associated with AD risk in IGAP (*p* = 0.0058). Two loci (rs15205, *p* = 0.019; rs79099429, *p* = 0.034) are significant for rate of decline (see “Data and methods for Rate of Progression Analyses” in Methods). rs15205 is a *ptau/Aβ42* rQTL (Table 2) involved in a *ptau* G×G interaction and rs79099429 is a *ptau/Aβ42* rQTL (Table 2). Plots similar to Fig. 1 for each interaction in Table 3 can be found in Additional file 1 (Figures S1–S3) along with brief comments. Further annotations for the variants in Tables 2 and 3 can be found in Additional file 2 (Tables S1 and S2).

## Discussion and conclusions

From our analyses, we have found evidence for rQTL that modulate the relationship between AD biomarkers. Furthermore, we have found that some of these rQTL interact with other loci (G×G), and some of these interactions directly contribute to the rQTL pattern. Ultimately, some of these loci affect the relationship between these biomarkers and AD and also directly affect the risk for AD and rate of decline.

The most intriguing result is the rs1036819 *ptau/*Aβ42 rQTL on chromosome 8 (Table 2), which is the second most significant rQTL found in our study. In follow-up a-priori tests, there is strong evidence that rs1046819 is also an rQTL for Aβ42 and AD case/control status and an rQTL for *tau/*Aβ42. This means that rs1036819 (or something associated with it nearby) modifies the relationship between Aβ42 and *ptau*, *tau*, and AD risk. In the separate and much larger AD risk dataset, rs1036819 is genotypically significantly associated with AD case/control status (*p* = 1.95 × 10^−5^) and there is a mild (but not significant) suggestion that it may impact the rate of decline (*p* = 0.0685). However, rs1036819 is not associated with case/control status in IGAP. It may be associated in ADGC because the CSF samples represent a higher concentration of samples in ADGC (~ 5.5%) than IGAP (~ 3%) or because ADGC represents a more homogeneous sample from populations more closely matching the CSF AD biomarker samples. The *ptau/*Aβ42 rQTL pattern shows a moderate negative covariate-corrected correlation between *ptau* and Aβ42 in the common AA and AC genotypes (−0.169 and −0.19), but a dramatically stronger negative correlation in the rare CC homozygote (−0.414). This appears to mirror the within genotype relationships between AD case/control status and Aβ42 where the rare homozygote has a much stronger negative relationship. The rare homozygote is also what drives the direct association with AD case/control status in the much larger independent dataset.

It is not entirely clear why rs1036819 or the region around it is important; however, it is a very strong GWAS hit for pelvic organ prolapse along with the most significant rQTL (rs8027714) in the study [35]. We are not suggesting that pelvic organ prolapse is related to AD or to the *ptau/*Aβ42 relationship; instead, this suggests that rs1036819 and rs8027714 may share some biological and potentially disease-related pathway. rs1036819 lies in a noncoding RNA (ncRNA) in an intron of the *ZFAT* gene and has a RegDB score of four, binds to the POL2 and POL24H8 proteins, and alters a Foxf2 regulatory motif. *ZFAT* is related to autoimmune thyroid disease [36]. This is of interest since various papers have asserted a relationship between the immune system and AD [37–39]. In addition, rs8027714 also interacts (G×G) with another locus for Aβ42 (Table 3, Fig. 1).

Another interesting finding involves the *tau/*Aβ42 rQTL, rs74025622. It interacts with three different loci for *tau* (Table 3, Additional file 1: Figure S2). It is upstream (5’) of a large GWAS hit for sphingolipids in the *CNTNAP4* gene, which has a role in neurotransmission of the dopaminergic and GABAergic systems, and mutations may be related to certain psychiatric illnesses. One particular locus it interacts with, rs9817620, is in the *CHL1* gene. The G×G is genome-wide significant for *tau*, has a nominally significant interaction for Aβ42 (*p* = 0.0013), and the pattern of interaction differs for the two traits in a way that creates the rQTL pattern of tau*/*Aβ42 correlation changes across genotypes (see Fig. 1a). *CHL1* is a neural cell adhesion molecule and may be involved in signal transduction pathways [40]. *CHL1* is related to *BACE1* as a substrate [41], which is a β-secretase enzyme [42] that initiates production of the β-amyloid peptide involved in AD and is a primary drug target [43]. *BACE1*-deficient mice showed phenotypic similarities to mice with CHL1 loss of function [44].

Only two of the 10 rQTL and one of the second G×G loci show any nominal direct association (i.e., a marginal/mean effect) with either of the traits in the rQTL or G×G models. This follows a trend where rQTL usually do not (but can) show a direct marginal (mean) effect with any of the traits in the rQTL making them invisible to typical association studies [5]. Theoretically, rQTL are often due to interactions with other loci or environments [6]. Specifically, differential epistasis occurs when the pattern of interaction between two loci differs across traits leading to trait1/trait2 correlation heterogeneity across the genotypes of one or both of the interacting loci. While interactions were not found for all rQTL, a number were found and, of those, many had a pattern of interaction with the two traits that directly contribute to the rQTL pattern (see Fig. 1 and Additional file 1: Figures S1–S3).

The greatest limitation of this study is the lack of an appropriate CSF biomarker study for replication of the rQTL and G×G. The purpose of this work is to generate hypotheses for future, large CSF biomarker studies to test. Consequently, we are not making grand assertions about our findings and our group is actively working to produce and assemble another CSF dataset that can be used for validation in the future. In fact, we are in a similar situation to the original authors of the recent paper [29] that performed the first analysis of the rate of progression in the ADNI and Knight ADRC data. They did not have a validation set and stated that their findings were meant to create hypotheses for future studies. Here we are using a small subset of their data and methods to connect our findings to make meaningful connections to disease progression. Subsequently, we have shown that some of these loci directly relate to AD through associations with AD risk and with rate of progression.

While context-dependent effects are hard to detect and to interpret, they are expected in biological systems and can help explain some of the heterogeneity among individuals. One hope is that with interactions we can identify subgroups that act differently from the “norm” and that these “exceptions” may help us to understand the more general responses or lead to different biological pathways.

## Additional files

Additional file 1: Additional rQTL and interaction plots. Figures S1, S2, and S3 show plots for each significant G×G along with their accompanying rQTL corresponding to Table 3 in the text and similar to Fig. 1 in the main text. (PDF 297 kb)

Additional file 2: Additional annotations for rQTL and G×G SNPs. Contains additional annotations for the rQTL in Table 2 and G×G SNPs in Table 3 in the main text. (XLSX 12 kb)

## Abbreviations

Aβ: Amyloid-beta
AD: Alzheimer’s disease
ADGC: Alzheimer’s Disease Genetic Consortium
ADNI: Alzheimer’s Disease Network Initiative
ADRC: Alzheimer’s Disease Research Center
CDR: Clinical Dementia Rating
CSF: Cerebrospinal fluid
DF: Degrees of freedom
G×E: Gene-by-environment
G×G: Gene-by-gene
GWAS: Genome-wide association studies
IGAP: International Genomics of Alzheimer’s Project
MAF: Minor allele frequency
rQTL: Relationship quantitative trait loci
SNP: single nucleotide polymorphism
