# Supplementary material for: Genome-wide association study for variants that modulate relationships between cerebrospinal fluid amyloid-beta 42, tau, and p-tau levels
Source: Alzheimers Res Ther. 2018 Aug 28;10:86. doi: 10.1186/s13195-018-0410-y (PMC6114488; doi:10.1186/s13195-018-0410-y)
Supplement: Supplementary file 1 — Additional rQTL and interaction plots. Figures S1, S2, and S3 show plots for each significant G×G along with their accompanying rQTL corresponding to Table 3 in the text and similar to Fig. 1 in the main text. (PDF 297 kb) [file 13195_2018_410_MOESM1_ESM.pdf]

Figure Legend

Figure S1. Each column has rQTL plot (bottom) and GxG plots for both traits above it. The rQTL plot displays the covariate-adjusted correlation between the two traits by genotype. For a particular locus that interacts with the rQTL, the GxG plots display the covariate adjusted two-locus genotypic means and error bars from bootstrapping. The first column of plots for the rs8027714 *tau*/Aβ42 rQTL (Table 1) on the bottom and its interaction with rs57134082 for Aβ42 (Table 2) and *tau*. The second column of plots for the rs689167 *ptau*/Aβ42 rQTL (Table 1) on the bottom and its interaction with rs1558634 for *ptau* (Table 2) and Aβ42. In all plots the top interaction is genome-wide significant (how it was found) and plotting the interaction for the second trait in row two is to describe the pattern. Both interaction shows the appearance of a dominance-by-dominance type of interaction and direction of heterozygote dominance is reversed in the two traits but it is not as clear whether this interaction actually relates to the rQTL pattern.

Figure S2. Plots for the rs74025622 *tau*/Aβ42 rQTL (Table 1) and interactions (Table 2) with three other loci (rs9817620, rs73105331, and rs75034965) similar to Figure S1. The three loci were found due to genome-wide significant interactions with rs74025622 for *tau*; however, rs9817620, rs73105331 also have nominally significant interactions with rs74025622 for Aβ42 (both have a p-value ~0.001). All three interactions fit the rQTL pattern where the crossing interactions are in the opposite direction for the two traits leading to greater negative correlation between the two traits in the heterozygote compared to the common homozygote.

Figure S3. The first column of plots for the rs79099429 p*tau*/Aβ42 rQTL (Table 1) on the bottom and its interaction with rs79688703 for *ptau* (Table 2) and Aβ42. The second column of plots for the rs15205 *ptau*/Aβ42 rQTL (Table 1) on the bottom and its interaction with

rs79688703 for *ptau* (Table 2) and Aβ42. These two interactions are effectively the same interaction. While the interaction is genome-wide significant for *ptau*, they also have a nominally significant interaction for Aβ42 (p~0.002) and have a nominal direct association with Aβ42 (p=0.016). The interactions also have opposite crossing patterns for the two traits leading to a greater negative correlation between the two traits in the heterozygote seen in the rQTL.

Figure S1.

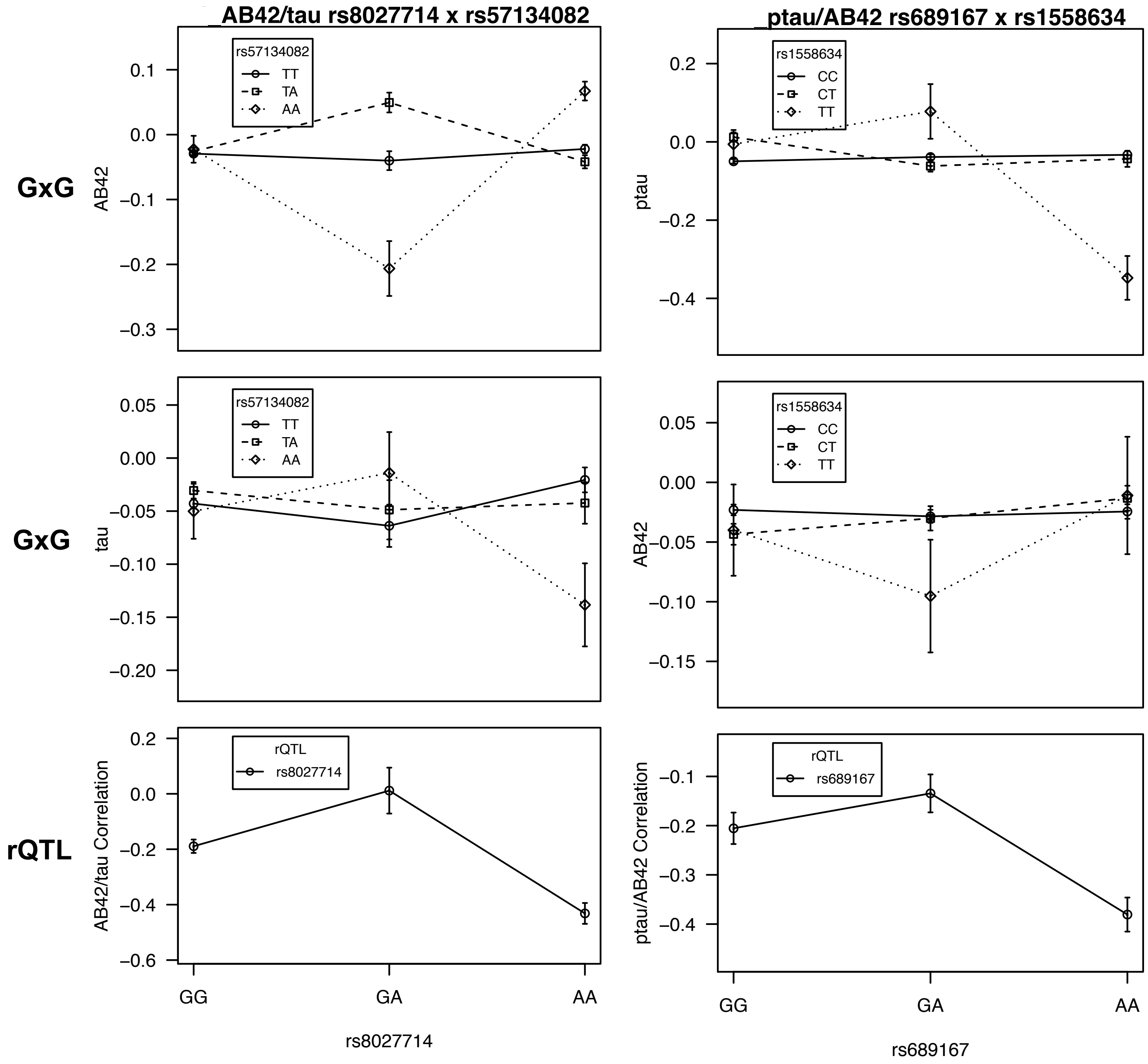
_AB42/tau rs8027714 x rs57134082
_ptau/AB42 rs689167 x rs1558634
GxG
GxG
rQTL
rs57134082
TT
TA
AA
rs1558634
CC
CT
TT
AB42
ptau
tau
AB42
rQTL
rs8027714
rQTL
rs689167
AB42/tau Correlation
ptau/AB42 Correlation
GG
GA
AA
rs8027714
rs689167

Figure S2

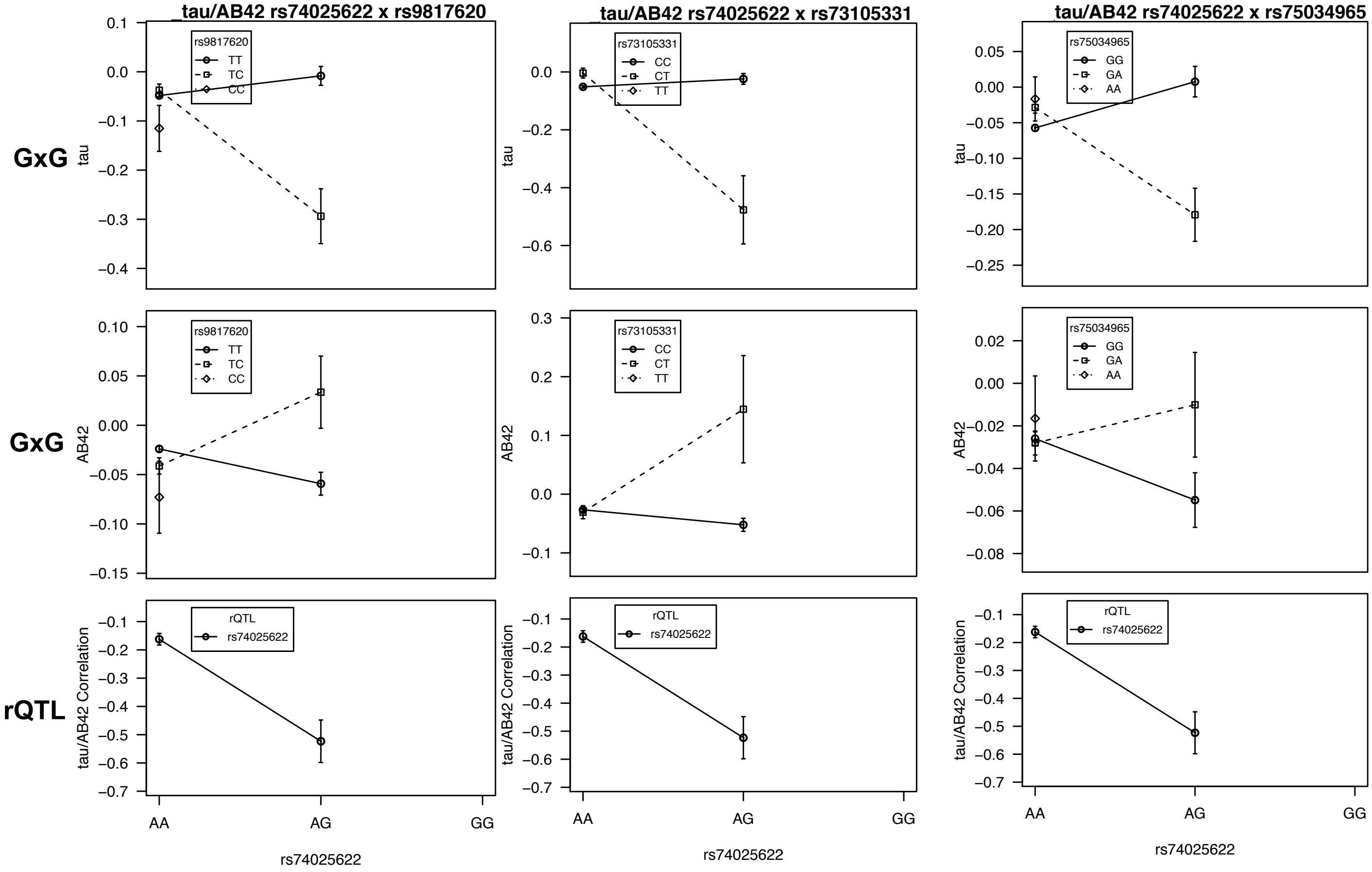

_tau/AB42 rs74025622 x rs9817620
_tau/AB42 rs74025622 x rs73105331
_tau/AB42 rs74025622 x rs75034965
GxG
GxG
rQTL
tau
AB42
tau/AB42 Correlation
rs9817620
TT
TC
CC
rs73105331
CC
CT
TT
rs75034965
GG
GA
AA
rQTL
rs74025622
AA
AG
GG
rs74025622

Figure S3

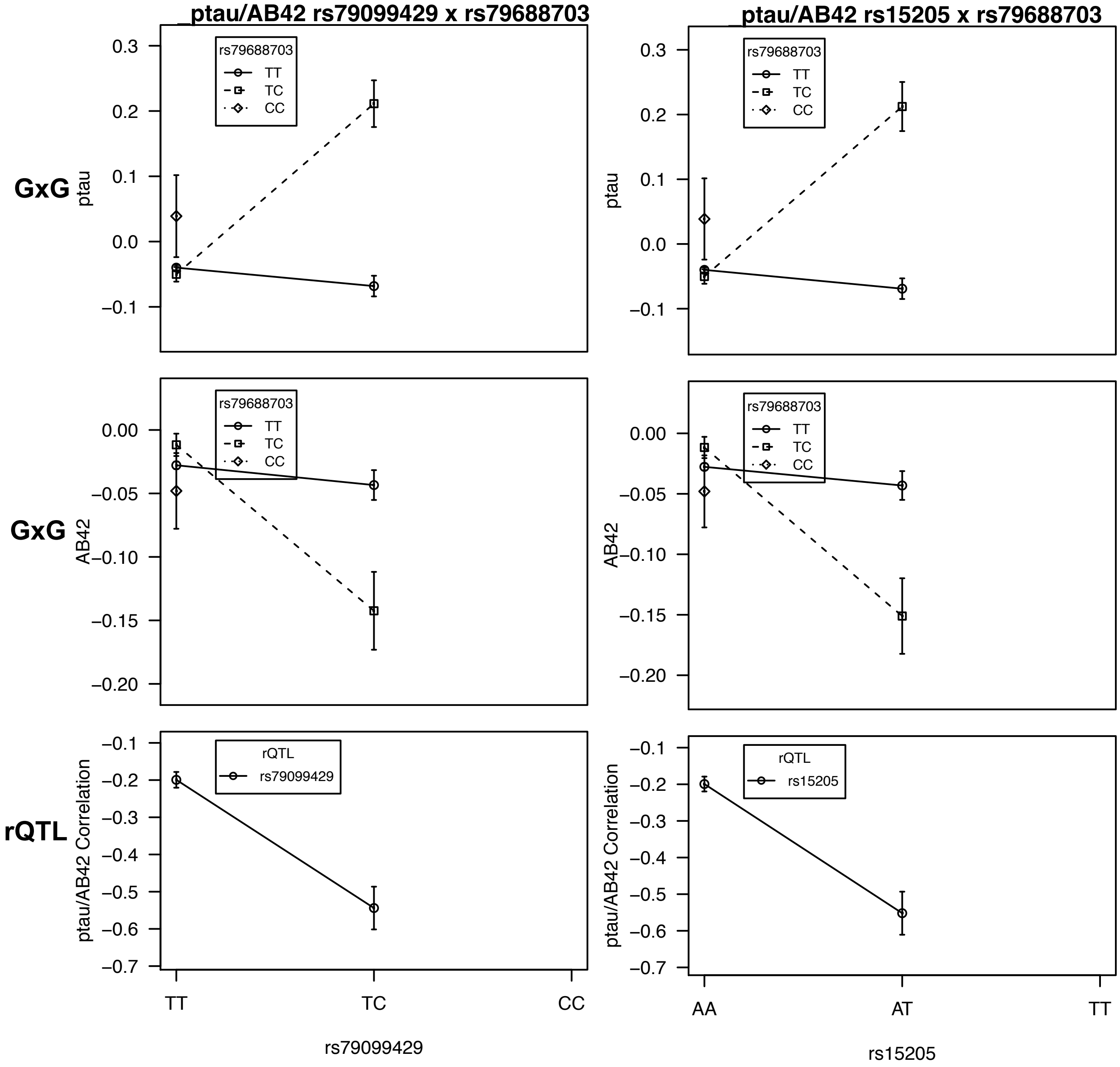

_ptau/AB42 rs79099429 x rs79688703
_ptau/AB42 rs15205 x rs79688703
GxG
GxG
rQTL
rs79688703
TT
TC
CC
ptau
AB42
ptau/AB42 Correlation
rQTL
rs79099429
rs15205
TT
TC
CC
AA
AT
TT
